# Extracellular LDLR repeats modulate Wnt signaling activity by promoting LRP6 receptor endocytosis mediated by the Itch E3 ubiquitin ligase

**DOI:** 10.18632/genesandcancer.146

**Published:** 2017-07

**Authors:** Sapna Vijayakumar, Guizhong Liu, Huei-Chi Wen, Yaa Abu, Robert Chong, Horacio Nastri, Gadi G. Bornstein, Zhen-Qiang Pan, Stuart A. Aaronson

**Affiliations:** ^1^ Oncological Sciences, Icahn School of Medicine at Mount Sinai, New York, NY, USA; ^2^ Princeton University, Princeton, New Jersey, USA; ^3^ Centers for Therapeutic Innovation, Pfizer Inc. New York, NY, USA; ^4^ Present address: Pfizer, San Diego, CA, USA; ^5^ Present address: China Suzhou Industrial Park, China; ^6^ Present address: Dermatology, Icahn School of Medicine at Mount Sinai, New York, NY, USA; ^7^ Present address: Ragon Institute of MIT, MGH, and Harvard, Cambridge, MA, USA; ^8^ Present address: Antibody Biotherapeutics, Incyte Corporation, Wilmington, DE, USA; ^9^ Present address: TESARO, Waltham, MA, USA

**Keywords:** Wnt, LRP6, LDLRR, sarcoma, Itch E3 ubiquitin ligase, internalization

## Abstract

The LOW-density lipoprotein related protein 6 (LRP6) receptor is an important effector of canonical Wnt signaling, a developmental pathway, whose dysregulation has been implicated in various diseases including cancer. The membrane proximal low-density lipoprotein (LDL) receptor repeats in LRP6 exhibit homology to ligand binding repeats in the LDL receptor (LDLR), but lack known function. We generated single amino acid substitutions of LRP6-LDLR repeat residues, which are highly conserved in the human LDLR and mutated in patients with Familial Hypercholesteremia (FH). These substitutions negatively impacted LRP6 internalization and activation of Wnt signaling. By mass spectrometry, we observed that the Itch E3 ubiquitin ligase associated with and ubiquitinated wild type LRP6 but not the LDLR repeat mutants. These findings establish the involvement of LRP6-LDLR repeats in the regulation of canonical Wnt signaling.

## INTRODUCTION

Wnt/β-catenin signaling is critical in embryonic development, and aberrations of this pathway play important roles in a variety of human diseases including cancer [1, 2]. β-catenin serves as a major Wnt transcriptional effector, whose functions are regulated by casein kinase 1α (CK1α) and glycogen synthase kinase 3β (GSK3β) phosphorylation, which target it for proteosomal degradation by beta-transducing repeat-containing protein (β-TrCP) in the absence of Wnt ligand stimulation [3-5]. When a canonical Wnt ligand binds to its cell surface co-receptors, LRP5/6 and Frizzled (Fz), phosphorylation of LRP6 occurs at multiple residues, including serine1490 and threonine1479 mediated by GSK3β [6] and CK1γ [7] respectively, leading to LRP6 aggregation at the plasma membrane [8]. In addition to LRP6, these aggregates or signalosomes contain several other Wnt pathway components including Axin, Dishevelled (Dvl), Frizzled (Fzd) and GSK3β, as well as caveolin, a marker of caveolin enriched membrane regions [8]. Signalosomes are subsequently internalized, leading to inhibition of β-catenin proteosomal targeting and its accumulation and transfer to the nucleus, where it acts as a co-transcription factor to increase transcriptional output of the T-cell factor (TCF)/lymphoid enhancer factor (LEF) family of transcriptional factors [1, 8, 9].

While evidence supports a critical role for LRP6 internalization in Wnt/β-catenin signaling [10-12], the exact mechanism of Wnt induced LRP6 internalization is not yet known. Several independent studies have shown that the caveolin endocytic pathway plays a critical role in Wnt mediated LRP6 internalization [10, 13]. A recent study found RAB8B, a Rab GTPase, to be required for caveolin mediated LRP6 endocytosis [13]. Down regulation of RAB8B inhibited Wnt signaling in cell culture, and knock down of RAB8B with morpholinos resulted in developmental delays in Xenopus embryos. A tetrameric sequence, Asn-Pro-Val-Tyr (NPxY), in the cytoplasmic region is implicated in the internalization of several cell surface receptors including the LDLR [14]. This sequence is absent in LRP6, and it is not known whether there is any specific LRP6 motif or sequence whose modification in response to Wnt, induces assembly of the endocytic machinery to internalize the LRP6 receptor complex and activate the Wnt/β-catenin pathway.

The extracellular region of LRP6 consists of four YWTD-type β-propeller domains, each interspersed by an epidermal growth factor (EGF)-like repeat domain (E1-E4), and three membrane proximal LDLR-type A domains [15]. The LDLR-type A repeats are about 40 amino acid residues in length and show about 50% to 70% similarity to those within the LRP5 receptor. Both functional and crystal structure studies have shown that Wnt ligands and antagonists bind to the β-propeller and EGF-like domains, while the LDLR repeats (LDLRR) appear to be dispensable [16-21]. Yet, mutational studies have indicated that the presence of LDLRR in N-terminally truncated LRP6 activates Wnt signaling much more strongly than LRP6 devoid of the entire ectodomain, supporting the functional importance of this domain in Wnt signal transduction [16-18]. Furthermore, a study previously showed that the LDLR repeats adversely affect LRP6 function by recruiting a negative regulator, Cdo, a multifunctional receptor protein [22].

In the present study, we analyzed the function of the LRP6 LDLRR by mutational approaches and showed that substitutions of LRP6 LDLRR amino acids, which are highly conserved with residues in the LDLR causing familial hypercholesterolemia (FH), inhibited LRP6 internalization and Wnt signaling activity. From mass spectrometric analyses to identify protein complexes recruited by the LRP6 wild type (WT) but not LDLRR mutants, we found that the E3 ubiquitin ligase, Itch, specifically co-immunoprecipitated with LRP6-WT. These and our other findings demonstrate that Itch ubiquitinates LRP6 and modulates its function in Wnt signal transduction.

## RESULTS

### Amino acid substitutions in the LRP6 LDLRR domain with disease association in the LDLR inhibit Wnt activity

**Figure 1:**
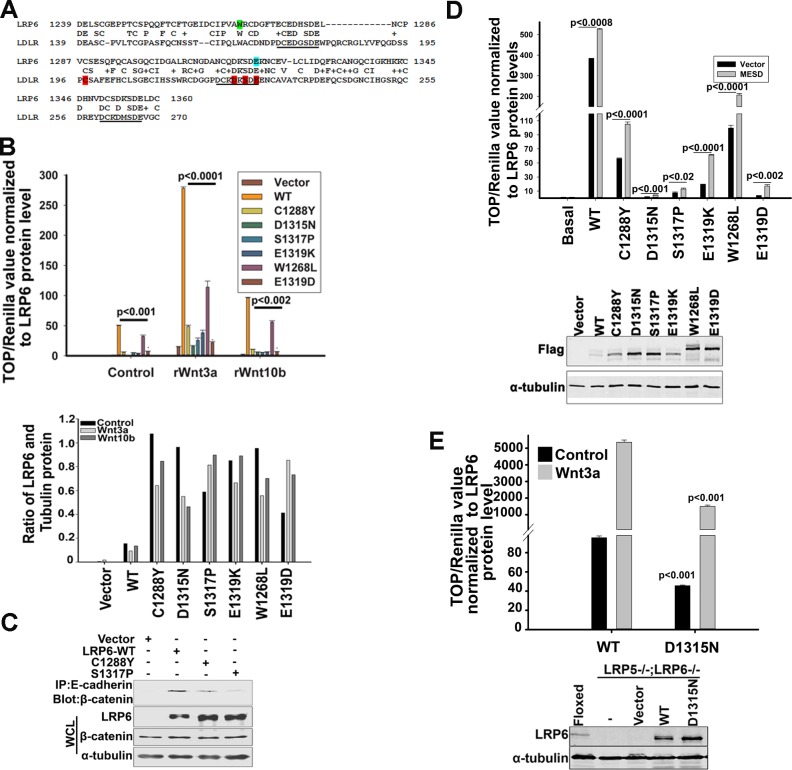
Effects of LDLRR point mutations on LRP6 mediated Wnt signaling (A). Alignment of human LRP6 and LDLR LDLRR domains. Residues highlighted in red are the FH mutations used to generate LRP6-LDLRR mutants. A mutation generated in an independent conserved region is in green and a reported SNP in LRP6 is in blue. Calcium binding regions in the LDL receptor are underlined. (B). TCF reporter activity in 293T cells transiently expressing wild type or LRP6-LDLRR mutants following overnight treatment with Wnt3a (100ng/ml) or Wnt10b (400ng/ml). Each data point in the graph is mean value of triplicates. Error bars indicate standard error of the mean of triplicates. The graph is representative of three independent experiments. The p values were calculated using unpaired two-tailed t-test comparing values of each LDLRR mutant to WT values within Control, rWnt3a and rWnt10b groups. Relative quantification of the pixels (lower panel) obtained from Western blotting of the lysates used in luciferase assay. (C). Uncomplexed β-catenin protein levels in 293T cells transiently expressing LRP6-WT or two representative LRP6-LDLRR mutants. Briefly, cells were rinsed and lysed in NP-40 buffer and processed for immunoprecipitation with GST-E-cadherin/glutathione conjugated beads as explained under Experimental Procedures section. (D). TCF reporter activity in 293T cells transiently expressing wild type or LRP6-LDLRR mutants in the presence of chaperone protein MESD. TCF luciferase values were normalized to renilla luciferase and their ratios normalized to corresponding LRP6 protein levels. Each data point is mean value of triplicates. Error bars indicate standard error of the mean of triplicates. The graph is representative of three independent experiments. The p values were calculated using unpaired two-tailed t-test comparing values of MESD to Vector for each of the LDLRR mutants or WT LRP6. Western blots showing the expression of various LRP6-LDLRR mutants are shown below. W1268L and E1319D constructs also have GFP tags, hence migrate differently. (E). TCF reporter values normalized to renilla luciferase in LRP5−/−; LRP6−/− double knockout MEFs transduced with and stably expressing human LRP6 wild type or D1315N mutant treated with Wnt3a overnight (left panel). Ratios of TCF and Renilla luciferase values normalized to LRP6 protein levels in MEFs are shown. Western blot comparing expression levels of lentiviral-transduced LRP6-WT and D1315N mutant in LRP5−/−; LRP6−/− double knockout MEFs (right panel). Each data point in the graph is mean value of triplicates. Error bars indicate standard error of the mean of triplicates. The graph is a representative of three independent experiments. The p values were calculated using unpaired two-tailed t-test comparing values of Controls or Wnt3a to each other in the two groups. Western blots were processed using Photoshop to adjust brightness/contrast and cropped to show all important bands.

To elucidate the function of LRP6-LDLRR, we initially used the basic local alignment tool (BLAST) to compare it with the human LDLR. We focused on amino acid sequences from 139 to 270 encoded by exons four and five of the LDLR that showed highest homology to the LRP6-LDLRR sequence (Figure 1 A). Within this 122 amino acid span in the LRP6-LDLRR (1239-1360), we identified previously reported point mutations in the LDLR that are causative of FH [23] (Table 1). Four of the missense mutations that decreased LDLR activity by more than 98% [23] were also conserved in both LRP6-LDLRR and LRP5-LDLRR (Table 1). We hypothesized that these conserved residues might also serve critical functions in LRP6 and, thus, used site-directed mutagenesis to create these amino acid substitutions in LRP6. Figure 1B shows that none of the FH mutants activated the TCF reporter, which measures Wnt signaling [24], as effectively as wild type LRP6. Furthermore, in cells expressing two representative FH mutants, activation of the Wnt pathway as measured by uncomplexed β-catenin protein levels, was lower than in cells expressing LRP6-WT (Figure 1C). We also created a W1268L substitution conserved between LRP6-LDLRR and LDLR but not reported as an LDLR disease mutation. W1268L retained activity comparable to wild type LRP6 (Figure 1B).

**Table 1:** List of FH mutations and the activity of LDL receptor in a region

LDLR	% Activity	Conserved on LRP6
E119K	15-30%	1240
C134G	15-30%	1256
D147H	<2%	1271
C152R	5-15%	1276
D154N	NR	1278
S156L	<2%	1280
C176F	<2	1288
C176Y	<2	1288
D200G	<2	1322(LRP5)
D203N	<2	1315
D203G	5-15	1315
S205P	<2	1317
D206E	5-15	1318
E207Q	2-5	1319
E207K	<2	1319
C227F	<2%	1338
D235G	5-15%	1346
D245E	15-30%	1356
C249Y	5-15%	1360

To address whether the decreased activity of LRP6-LDLRR mutants might be due to protein misfolding, we coexpressed mesoderm development candidate 2 (MESD), a chaperone involved in LRP6 receptor maturation [25, 26]. The presence of MESD partially rescued the activities of C1288Y and E1319K, albeit not to the level of LRP6 wild type (Figure 1D). Coincidently, co-expression of MESD also increased total LRP6 protein levels in cells expressing LRP6-WT and C1288Y (data not shown). MESD had no effect on the Wnt signaling activities or total LRP6 protein levels of other FH mutants, indicating that misfolding was unlikely to be the basis for their decreased function. Finally, we investigated the effects of point mutations in the LRP6 LDLRR in a LRP5/6 null background using mouse embryo fibroblasts (MEFs) engineered to lack both LRP5 and LRP6 [27]. Figure 1E shows that a mutant with a conserved FH substitution exhibited much lower TCF reporter activity compared to LRP6-WT at similar protein expression levels. Together, the above findings argue that the LDLRR domain contributes importantly to LRP6 Wnt signaling functions.

### Endocytosis of LRP6 requires an intact LDLRR domain

**Figure 2:**
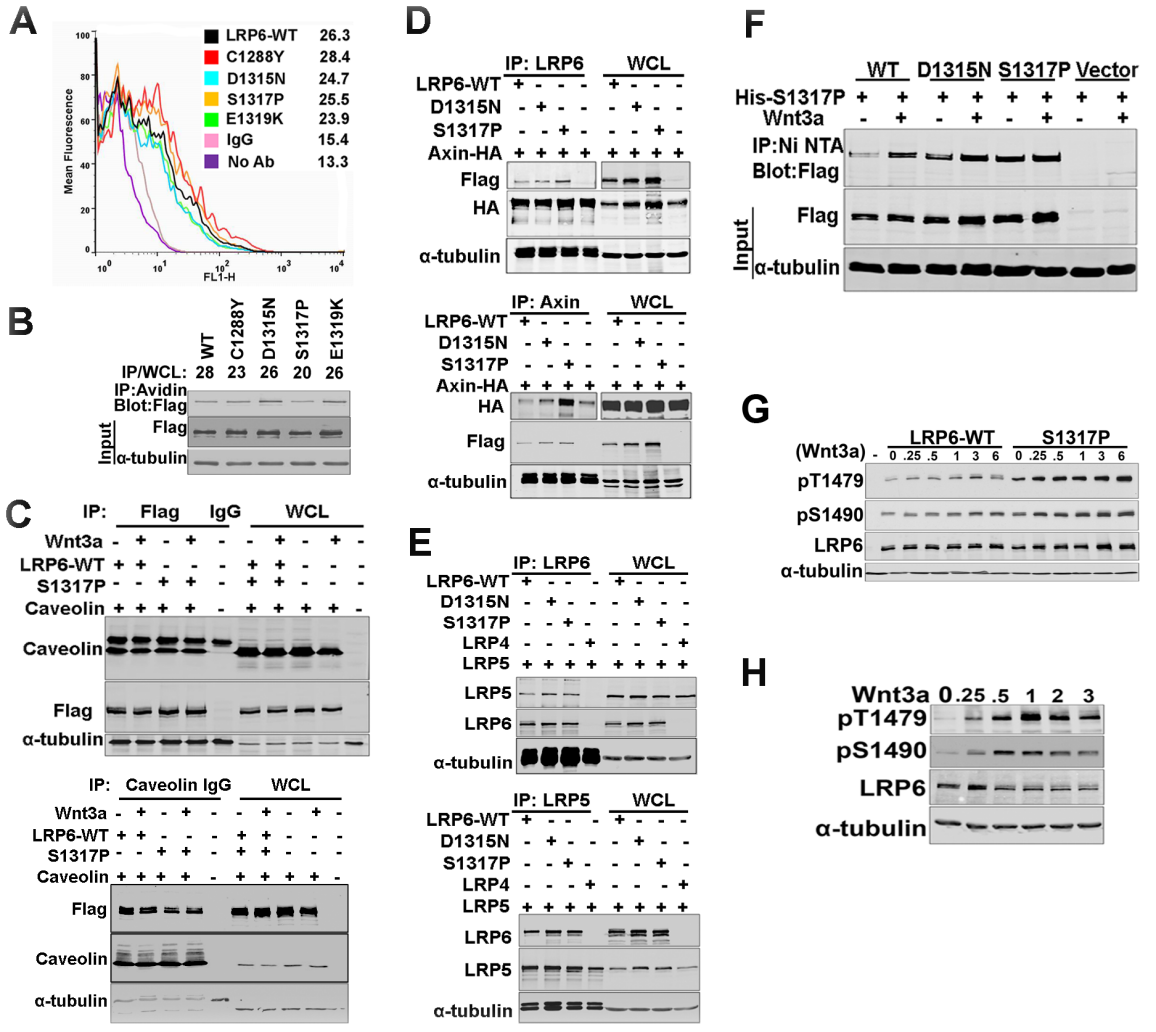
Comparison of early Wnt signaling events and engagement of downstream pathway components by LRP6 wild type and LDLRR mutants (A). Cell surface expression of LRP6 WT or LDLRR mutants in 293T cells. Briefly, 48hrs following transient transfection with flag-tagged LRP6 constructs, cells were fixed with 4% paraformaldehyde and stained with flag antibody followed by a fluorescently labeled secondary antibody and processed for fluorescent activated cell sorting analysis (FACS). The values represent geometric mean obtained from 10,000 events counted. (B). Cell surface expression of LRP6 WT or LDLRR mutants in 293T cells. 293T cells transfected with either WT or LDLRR mutant LRP6 were labelled with cell impermeable biotin. Biotin was then removed, and cells were rinsed, lysed in NP-40 buffer and processed for IP with avidin-conjugated beads. Protein eluted was used for immunoblotting (top). The values for IP/WCL indicate pixel ratios obtained from Flag in the IP normalized to Flag/Tubulin values in the whole cell lysates (WCL). (C). Immunoprecipitation (IP) of Flag tagged LRP6 WT or S1317P mutant from 293T cells co-expressing caveolin 48hrs after transient transfection and exposure to Wnt3a for one hour (top). Following IP with Flag beads, proteins were eluted and used for immunoblotting. The membrane was probed with caveolin, Flag or α-tubulin. Reverse IP with caveolin using the same whole cell lysates (bottom). (D). Lysates from 293T cells co-expressing Flag tagged LRP6 WT or LDLRR mutants and HA tagged Axin were subjected to IP with anti LRP6 antibody [57] (top) or axin antibody (bottom). The HA band in the “no” LRP6 IP reflects exogenous HA-Axin co-precipitated with endogenous LRP6. (E). 293T cells co-expressing LRP6-WT or LDLRR mutants with LRP5-WT or LRP4-WT were lysed and processed for IP with LRP6 (D: top) or LRP5 antibodies (D: bottom). (F). 293T cells co-expressing flag tagged LRP6 WT or LDLRR mutants and his-tagged LRP6 S1317P mutant were processed for IP with nickel NTA column. Following immunoblotting, the membrane was probed with Flag antibody. (G). 293T cells expressing LRP6-WT or S1317P LDLRR mutant were treated with 100ng/ml of Wnt3a for the times (in hours) indicated and processed for immunoblotting. Membranes were probed with phospho-specific antibodies against LRP6 S1490 and T1479 or total LRP6. (H). 293T cells were treated with Wnt3a for the times (in hours) indicated and processed for immunoblotting. Membranes were probed with phospho-specific antibodies against LRP6 S1490 and T1479 or total LRP6. The band observed in the IP samples for tubulin is mouse IgG heavy chain. In each case, representative results from at least two independent experiments are shown. Western blots were processed using Photoshop to adjust brightness/contrast and cropped to show all important bands.

In an effort to identify the molecular defect in LRP6-LDLRR mutants, we performed flow cytometric analyses, which revealed that the mutants were comparably expressed at the cell surface relative to LRP6-WT (Figure 2A). Moreover, biochemical cell surface localization using biotinylated proteins also showed comparable protein expression of LRP6-WT and LRP6-LDLRR mutants (Figure 2B). It has been reported that caveolin enriched membrane localization of LRP6 is critical for efficient activation of Wnt signaling [10]. Both LRP6-WT and a representative LRP6-LDLRR mutant, S1317P, which was one of the least active in Wnt reporter assays (Figure 1B), immunoprecipitated with caveolin, a component of caveolin enriched membrane regions [28] (Figure 2C). A critical step after Wnt binding to LRP6 is the recruitment of Axin to membrane bound LRP6 [6, 7]. We did not observe any differences in the abilities of two of the least Wnt active FH mutants, D1315N and S1317P (Figure 1B), or LRP6-WT to engage Axin as shown by immunoprecipitation (Figure 2D). In line with previous findings that LRP6 homo-dimerizes [17] and hetero-dimerizes with LRP5 [29], two representative FH mutants, D1315N and S1317P, formed heterodimers with LRP5 (Fig.2E) and homodimers with LRP6-WT or a different FH mutant (Figure 2F). Compared to LRP6-WT, we observed no significant differences in the phosphorylation kinetics of S1490 or T1479 residues [6, 7] of a representative FH mutant, S1317P, in response to exogenous Wnt3a (Figure 2G). At endogenous LRP6 protein levels, the kinetics of T1479 and S1490 phopshorylations in response to exogenous Wnt3a indicated peaks at 1 hr of Wnt3a treatment (Figure 2H). These data collectively show that LRP6-LDLRR mutants retained the ability to perform early activation steps involved in Wnt signaling including homo-and heterodimerization with LRP5/6.

**Figure 3:**
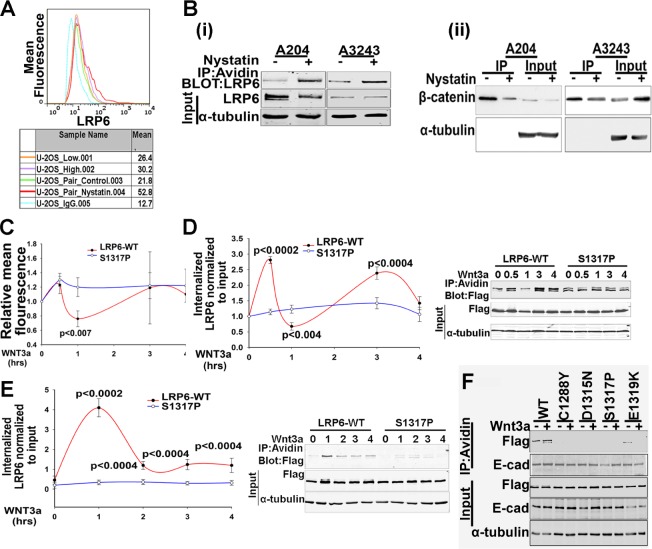
Comparison of Wnt ligand mediated internalization of LRP6 WT and a representative LDLRR mutant (A). Cell surface LRP6 expression in Wnt autocrine sarcoma cell line, U-2OS following Nystatin treatment. Low and High indicate cell confluency. For Pair Control and Nystatin, cell numbers were similar at the start of treatment. FACS analysis was performed as in Figure 2A, except that monoclonal LRP6 antibody [57] was used. The values represent the geometric mean obtained from 10,000 events counted. (B). Effect of Nystatin on cell surface LRP6 expression (i) and uncomplexed β-catenin level (ii) in Wnt autocrine A204 and A3243 human sarcoma cells [30]. Cells were pre-treated with Nystatin for 5 hours and then labelled with cell impermeable biotin. After incubation, cells were rinsed and lysed in NP-40 buffer and processed for immunoprecipitation with either Avidin conjugated beads or with GST-E-cadherin/glutathione conjugated beads. (C). 293T cells transfected with either WT or LDLRR mutant LRP6 were treated with Wnt3a for different times as indicated and then fixed in 4% paraformaldehyde and processed for FACS analyses as in Figure 2A. The mean fluorescence values obtained were normalized to the untreated sample (shown as 1). Each data point represents the mean value obtained from at least 3 independent experiments performed under similar conditions. Error bars indicate SEM. The graph is representative of three independent experiments. The p values were calculated using unpaired two-tailed t-test comparing values at each time point between the two groups. (D). Cell surface clearance of LRP6 in Wnt3a treated cells. 293T cells transfected with either WT or LDLRR mutant LRP6 were treated with Wnt3a for the times indicated. After rinsing, cells were labelled with cell impermeable biotin. Biotin was then removed, and cells were rinsed, lysed in NP-40 buffer and processed for IP with avidin-conjugated beads. Protein eluted was used for immunoblotting. The pixel values obtained for LRP6 from IP were normalized to those obtained for LRP6 in WCL and represented as relative ratios. Each data point is mean value obtained from at least three independent experiments run at similar conditions. Error bars indicate SEM. The graph is a representative of three independent experiments. The p values were calculated using unpaired two-tailed t-test comparing values at each time point between the two groups. (E). LRP6 internalized after Wnt3a treatment of 293T cells. This experiment was performed as in Fig.3D, except that cells were biotin labelled prior to Wnt3a treatment. Following Wnt3a treatment for the time intervals indicated, cells were rinsed and incubated with glutathione on ice to remove any remaining biotinylated cell surface proteins prior to lysis and IP. Each data point in the graph is mean value obtained from at least three independent experiments performed under similar conditions. Error bars indicate SEM. The graph is a representative of three independent experimental repeats. The p values were calculated using unpaired two-tailed t-test comparing values at each time point between the two groups. (F). Comparison of LRP6 internalized after 1 hr of Wnt3a treatment of 293T cells expressing WT or various LDLRR mutants. This experiment was performed as in Figure 3E. Western blots were processed using Photoshop to adjust brightness/contrast and cropped to show all important bands.

Several studies have recently shown that Wnt ligand binding induces LRP6 internalization through the caveolin-mediated pathway [10, 13]. Moreover, blocking this endocytic pathway using either RNAi against caveolin or Nystatin [10] inhibits Wnt signaling. Consistent with these reports, treatment of a Wnt autocrine human sarcoma cell line, U2-OS [30], with Nystatin increased cell surface LRP6 levels and simultaneously decreased uncomplexed β-catenin levels (Figure 3A). Furthermore, Nystatin also increased LRP6 cell surface levels in two other Wnt autocrine sarcoma cell lines, A204 and A3243, and decreased uncomplexed β-catenin protein levels in these cells (Figure 3B). To test whether LRP6-LDLRR mutants underwent ligand-mediated endocytosis, 293T cells expressing either LRP6-WT or a representative minimally Wnt active FH mutant, S1317P, were treated with Wnt3a for various periods and cell surface levels of LRP6 analyzed by flow cytometry. Figure 3C shows that within 1hr of Wnt3a treatment, cell surface LRP6-WT levels decreased by more than 20%, while there was no significant reduction in surface expressed S1317P mutant under the same conditions. We also compared cell surface levels of LRP6-WT and S1317P mutant using a cell surface biotinylation assay. Within 1hr of Wnt3a treatment, levels of biotinylated LRP6-WT were reduced by more than 30%, while there was no decrease in biotinylated S1317P levels (Figure 3D).

We next compared the abilities of LRP6-WT and the S1317P mutant to internalize in response to Wnt3a treatment. For this purpose, cells were pre-incubated with cell-impermeable biotin prior to Wnt3a addition and kinetics of internalization determined after exposing cells to glutathione. Only internalized biotinylated proteins are protected from glutathione cleavage and therefore, available for immunoprecipitation by avidin. Using this strategy, we found that the levels of internalized LRP6-WT increased by 1hr of Wnt3a treatment while S1317P mutant-expressing cells showed no detectable increase in internalized LRP6 levels under the same conditions (Figure 3E). Also, while LRP6-WT showed increased internalization upon 1hr of Wnt3a treatment, the LRP6-LDLRR mutants failed to internalize (Figure 3G). Taken together, these results suggest that mutations within the LDLRR domain inhibit Wnt3a-mediated endocytosis of LRP6. However, additional studies will be required to definitively establish that the observed LRP6 internalization defect is caveolin mediated.

### Itch E3 ubiquitin ligase mediated endocytosis is compromised in Wnt signaling defective LRP6 LDLRR mutants

**Figure 4:**
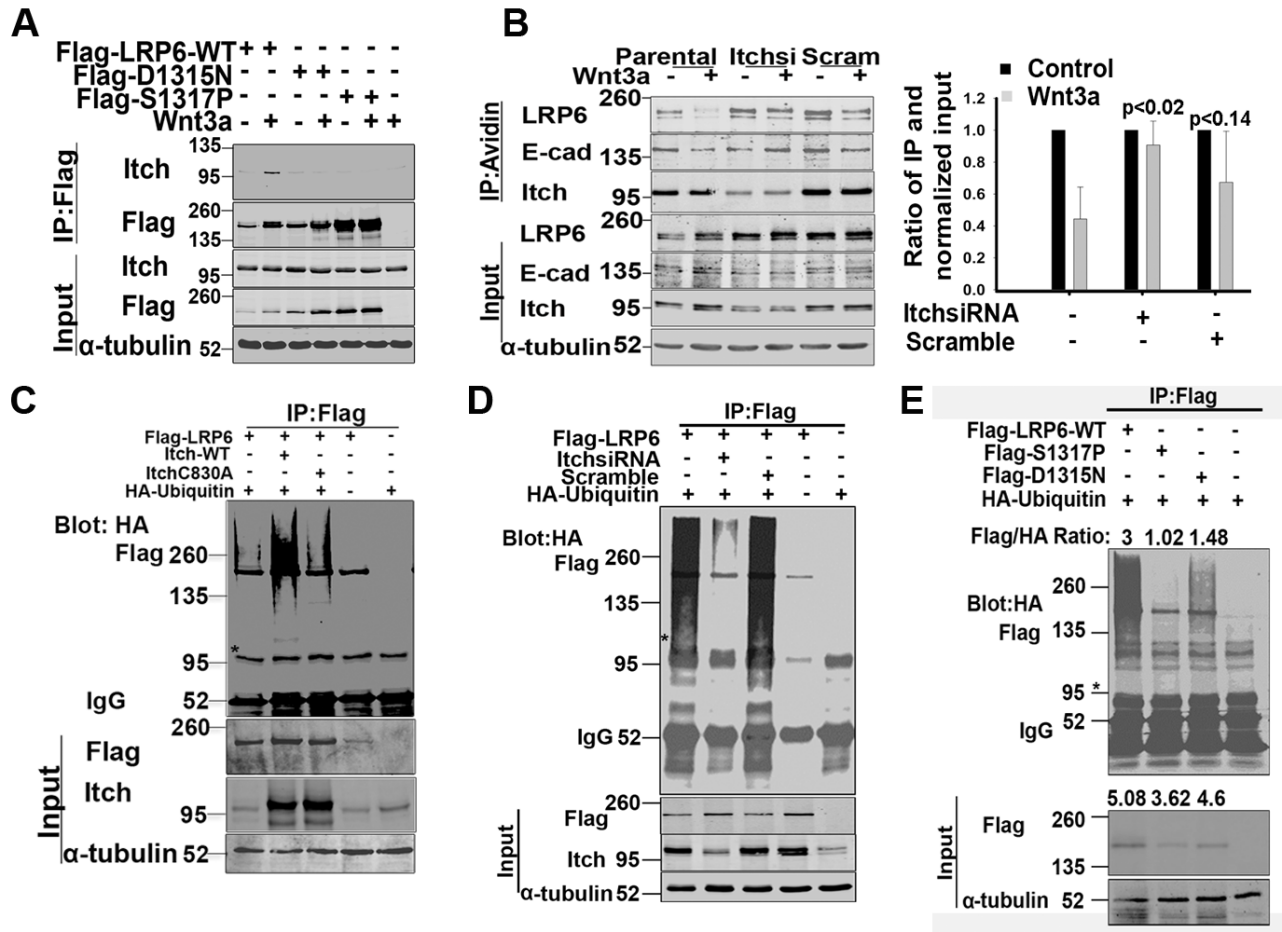
LRP6 ubiquitination by Itch E3 ligase (A). Itch interaction with LRP6. 293T cells transfected with LRP6 WT or LDLRR mutant were treated with Wnt3a for 1hr. Cells were then lysed using NP40 buffer and processed for IP with Flag antibody followed by immunoblotting. (B). Effect of Itch knock down on LRP6 internalization. 293T cells were transfected with either siRNA targeting Itch or control scramble siRNA. 48 hrs post-transfection, cells were labelled with cell impermeable biotin and then treated with Wnt3a for 1hr. Cells were processed for IP with avidin conjugated beads followed by immunoblotting as in Figure 3E. The bar graph was generated as in Fig.3D. Each bar presents data from one sample and error bars are SEM obtained from independent experiments run at similar conditions (n=3). The p values were calculated using unpaired two-tailed t-test comparing values of Wnt3a treated Itch and scramble siRNAs samples to Wnt3a treated parental sample. (C). Ligase active Itch ubiquitinates LRP6. 293T cells were co-transfected with flag tagged LRP6-WT and HA tagged ubiquitin and 24 hrs later trypsinized. Equal numbers of cells were replated and 24 hrs later transfected with Itch wild type or ligase dead ITCH C830A. At 40 hrs following the second transfection, cells were lysed in the presence of 1% SDS and processed for IP with Flag conjugated beads followed by immunoblotting. The same blot was probed sequentially with HA and flag using Licor secondary antibodies with either 700 or 800 spectra. *An unidentified band at 95kDa is seen. (D). Endogenous Itch ubiquitinates LRP6. 293T cells were co-transfected with HA tagged ubiquitin and flag tagged WT LRP6. Cells were trypsinized 24hrs post transfection, replated and transfected 24 hrs later with either Itch siRNA or scramble siRNA. 48hrs post second transfection, cells were processed as in Figure 4C. (E). Ubiquitination of LRP6-LDLRR mutants. 293T cells were transfected with HA tagged ubiquitin, and 24hrs post transfection were trypsinized and replated. 24hrs later, the cells were transfected with LRP6 WT or LDLRR mutant. Cells were processed for IP and immunoblotting as in Figure 4C. Flag/HA ratios are pixel values obtained from Flag IP normalized to HA from IP for each sample. In the WCL, the ratios are from Flag and tubulin. The blots shown are representative of independent experiments conducted for reproducibility (n≥2). Western blots were processed using Photoshop to adjust brightness/contrast and cropped to show all important bands.

To investigate the molecular mechanism responsible for inefficient endocytosis of the LDLRR mutants, we sought to identify differences between protein complexes recruited by them versus LRP6-WT. Mass spectrometric analyses were performed to identify and compare proteins that co-immunoprecipitated with wild type LRP6 and the two least Wnt active LDLRR mutants, S1317P and D1315N. We observed that the Itch E3 ubiquitin ligase (Itch) specifically co-immunoprecipitated with LRP6-WT in the presence of Wnt3a, but not with two representative LDLRR mutants (Figure 4A and Supplementary Table 1). To gain insights into the possible involvement of Itch in LRP6 internalization, we knocked down endogenous Itch in 293T cells and measured the effects on cell surface levels of endogenous LRP6. In the parental cells, Wnt3a treatment induced more than 50% cell surface clearance of LRP6 relative to the control cells (Figure 4B). Similar results were observed following Wnt exposure in cells treated with scramble siRNA. In contrast, cells expressing Itch siRNA showed no reduction in LRP6 cell surface protein levels under the same conditions (Figure 4B).

Ubiquitination by Itch has been shown to mediate the internalization and endosomal sorting of certain cell surface receptors and other membrane bound proteins [31-33]. To test whether Itch ubiquitinated LRP6, we co-expressed LRP6-WT either with Itch-WT or a ligase defective Itch C830A mutant [32]. Under denaturing conditions, Itch-WT but not Itch C830A increased LRP6 ubiquitination as evidenced by the increased level of LRP6 high molecular weight forms (Figure 4C). Moreover, knock down of endogenous Itch but not the scramble control siRNA decreased LRP6-WT ubiquitination (Figure 4D). When compared to LRP6-WT, LRP6-LDLRR mutants S1317P and D1315N showed reduced ubiquitination (Figure 4E), and Itch knock down did not alter their ubiquitination levels (data not shown). Often, ubiquitination of proteins is associated with their proteosomal degradation [34]. However, we did not observe either proteosomal or lysosomal degradation of LRP6 even when Itch was over expressed (data not shown). These results suggest that Itch mediated ubiquitination of LRP6 serves non-proteolytic functions. Collectively, these data suggest that the LDLRR domain contributes to Wnt mediated endocytosis of LRP6 by modulating its interaction with Itch.

### LRP6 ubiquitination enhances its endocytosis and Wnt signaling function

**Figure 5:**
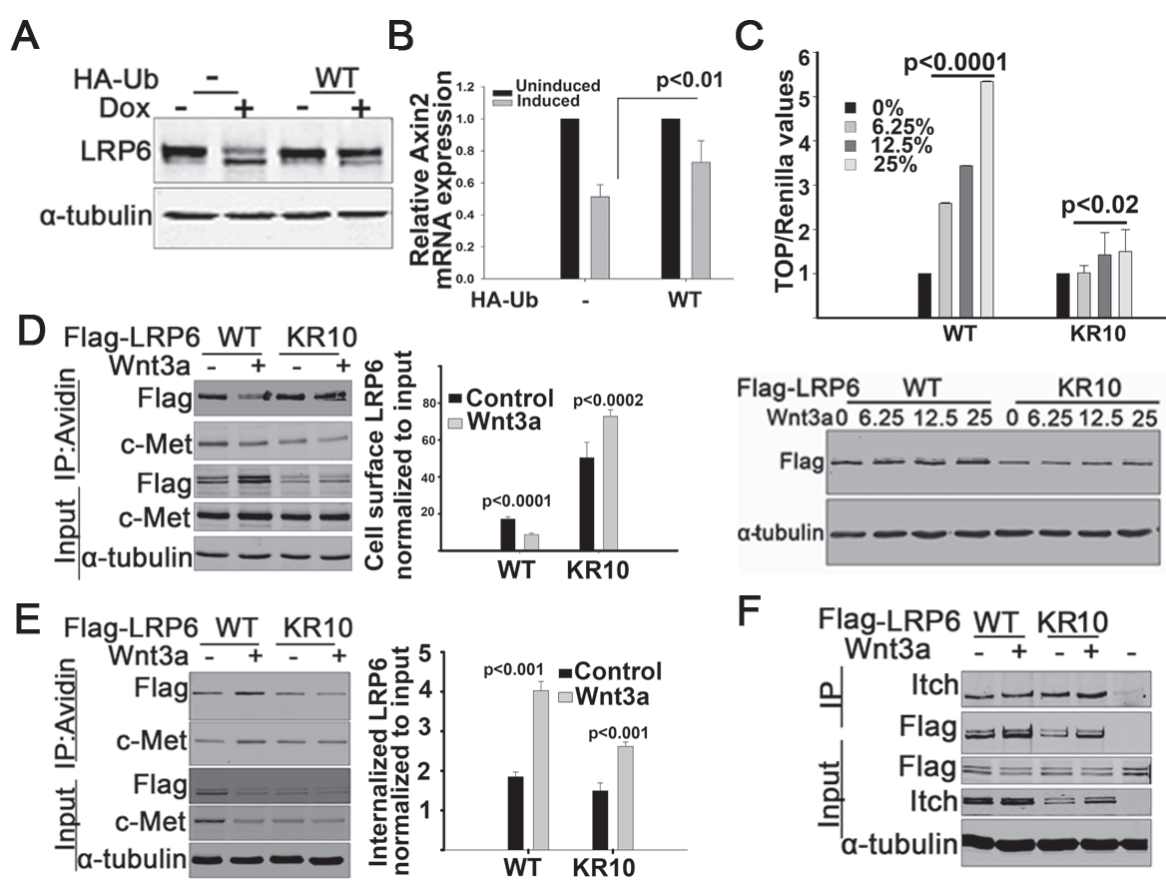
Role of LRP6 ubiquitination on Wnt3a mediated internalization (A&B). Endogenous LRP6 protein and Axin2 mRNA expression in ubiquitin knockdown or ubiquitin rescued Wnt autocrine U-2OS sarcoma cells. U-2OS cells engineered to be devoid of endogenous ubiquitin and expressing exogenous WT ubiquitin under Dox inducible promoter were used [35]. 72hrs post induction with Dox, cells were processed either for immunoblotting (A) or for real-time PCR (B). Axin2 mRNA expression was normalized to TATA Box Binding Protein (TBP). Each bar represents the mean of triplicates from one experiment, and error bars are SEM. The entire experiment was repeated twice with similar results. The p values were calculated using unpaired two-tailed t-test comparing values of “induced” in the two groups. (C). Wnt signaling activity of a cytoplasmic lysine mutant LRP6. All lysines present in the cytoplasmic tail of LRP6-WT were mutated to arginine, and the resulting mutant, designated KR10, or WT LRP6 was transfected in 293T cells stably expressing TCF and Renilla luciferase. 24 hrs later, the cells were treated with different concentrations of Wnt3a conditioned medium as indicated and processed for dual luciferase assay (top) or immunoblotting (bottom). Treated samples are represented relative to the control untreated sample in both WT and KR10 groups. Each bar represents the mean of triplicates from one experiment and error bars are SEM. The entire experiment was repeated twice. The p values were calculated using unpaired two-tailed t-test comparing values of treated samples with the control untreated in each group. (D). Cell surface clearance of cytoplasmic lysine mutant LRP6. 293T cells transfected with wild type or lysine mutant LRP6 were labeled with biotin, then treated with Wnt3a for 1 hr and processed as in Figure 3D. c-MET was used as a cell surface control protein. The graph on the right represents quantification of the pixels from the immunoblots as described in Figure 3D. Bars represent mean values of three independent experiments conducted under similar experimental conditions and error bars are SEM. The p values were calculated using unpaired two-tailed t-test comparing values of treated and untreated samples in each group. (E). Internalization of LRP6 KR10 mutant. 293T cells transfected with WT or KR10 were labeled with biotin, then treated with Wnt3a 1hr and processed as in Figure 3E. The graph was generated as in Figures 3E & 5D. (F). Interaction of the LRP6 KR10 mutant with Itch. 293T cells transfected with wild type Itch were replated and 24hrs later, transfected with LRP6 WT or KR mutant. 36hrs later the cells were treated with Wnt3a for 1hr and processed for IP and immunoblotting as in Figure 4A. Last lane:IP with IgG Control; All other lanes: IP with Flag The experiment was repeated twice with similar results. Western blots were processed using Photoshop to adjust brightness/contrast and cropped to show all important bands.

To gain further insights into the functions of LRP6 ubiquitin modifications induced by Itch, we used a doxycycline inducible HA-Ubiquitin expressing human osteosarcoma cell line, U2-OS, in which the expression of endogenous ubiquitin was replaced with HA tagged wild type ubiquitin [35]. Using this ubiquitin replacement system, we observed that cells in which endogenous ubiquitin was knocked down (Figure 5A), showed reduced levels of the higher molecular weight form of LRP6 (top band in LRP6 doublet) compared to the parental cells (Figure 5A). Exogenously expressed ubiquitin partially rescued this higher molecular weight LRP6 (Figure 5A). Previously, we reported that U-2 OS cells exhibit high Wnt signaling activity mediated by an autocrine mechanism [30]. When endogenous ubiquitin was knocked down in these cells, mRNA levels of Axin2, a direct Wnt transcriptional target gene [36], were reduced by about 50% compared to Axin2 mRNA levels in uninduced parental cells (Figure 5B). Moreover, Axin2 RNA levels were partially rescued by exogenous wild type ubiquitin expression (Figure 5B). All of these results suggest that the LRP6 ubiquitin modifications affect its Wnt signaling activity.

To independently investigate the effects of Itch ubiquitination on LRP6 function, an LRP6 mutant, in which all lysine residues in the cytoplasmic region were substituted with arginine, was tested in the TCF reporter assay. While there was a dose dependent increase in reporter activity in response to Wnt3a in cells expressing wild type LRP6, the mutant, designated KR10, exhibited significantly reduced reporter activity in response to Wnt3a (Figure 5C). We also observed that under these same conditions, KR10 was less well internalized compared to wild type LRP6 in response to Wnt3a (Figure 5D&E). As expected based on their wild type LDLRR domains, both wild type and lysine mutant LRP6 interacted with Itch to similar extents (Figure 5F). Together, these observations indicate that LRP6-WT binds to Itch and that Itch ubiquitination of its cytoplasmic domain positively influences both Wnt ligand mediated LRP6 internalization and signaling activity. In the case of the LDLRR mutants, Itch does not interact or ubiquitinate LRP6, thereby leading to their inability to internalize and activate downstream Wnt signaling.

## DISCUSSION

Canonical Wnt ligand binding to LRP5/6 and Fzd co-receptors triggers Dvl mediated plasma membrane associated LRP6 aggregation and signalosome formation [8]. Besides these Wnt components, signalosomes contain caveolin, a protein involved in the endocytic pathway. Although LRP6 endocytosis through caveolin plays a critical role in Wnt signaling [10], an unresolved question is the molecular mechanism by which LRP6 is targeted to caveolin mediated endocytosis. Our present study uncovers a new layer of LRP6 regulation influenced by its LDLRR domain, whose function has not previously been characterized. We showed that single amino acid substitutions of conserved residues between LRP6-LDLRR analogous to LDLR mutations in familial hypercholesterolemia (FH), impaired LRP6 ability to activate Wnt signaling. Furthermore, we provided evidence that the intact LDLRR region contributes to LRP6 interaction with the Itch E3 ubiquitin ligase and that the resulting ubiquitination of the LRP6 intracellular domain positively influences LRP6 endocytosis known to be required for activation of Wnt signaling. While studies with Nystatin suggest that LRP6 endocytosis may be mediated by caveolin, additional experiments will be required to definitively demonstrate that this is the case.

Unlike β-catenin and adenomatous polyposis coli (APC), both of which are frequently mutated in cancer [37], oncogenic mutations have not been observed in LRP6. However, mutations adversely affecting LRP6 function have been reported in early coronary artery disease (CAD) [38], and LRP6 loss of function has been linked to low bone density and multiple developmental defects in mice [39]. In CAD, a single amino acid substitution of cysteine for arginine at position 611 of LRP6 decreased Wnt ligand binding affinity and concomitantly, reduced its Wnt signal output [38]. Analysis of previously resolved crystal structures of the LRP6 ectodomain indicated that none of the point mutations we generated in the LRP6-LDLRR reside in a region essential for Wnt ligand or antagonist binding [20, 21]. In contrast, the FH mutations in the LRP6-LDLRR mutants generated are localized to the lipid binding domain in the LDLR and abrogate LDL/receptor engagement [23].

Based on the partially inactivating LRP5 C1351G mutation found in patients with familial exudative vitreoretinopathy (FEVR) [40], Chen et al. recently substituted glycine at the corresponding conserved cysteine residue in the LRP6-LDLRR, which resulted in reduced Wnt signaling activity [41]. This mutant appeared to have a decreased ability to undergo homodimerization [41], which is in contrast to the mechanism elucidated by us for the LDLRR mutants characterized here. In fact, our LDLRR mutations did not affect cell surface targeting of LRP6, its homotypic or heterotypic interactions with exogenous wild type LRP6 or LRP5 receptors, consistent with previous reports [17, 18], or inhibit the function of endogenous wild type receptors. In fact, previous studies have shown that its E1-E4 domains are primarily responsible for LRP6 dimerization and oligomerization [17]. Whereas we used full length LRP6 to generate LDLRR mutants, Chen at al. used an ECD mutant lacking E1–E4 domains, which may help to account for differences in results.

It is widely accepted that receptor endocytosis via clathrin coated pits or caveolin mediated caveolae enriched membrane regions play a key role in signal transduction by receptor tyrosine kinases [42], G-protein coupled receptors [43], TGF-β [44] and Notch [45] receptors [46]. There is also mounting evidence to support an essential role for endocytosis in the activation of Wnt signaling [12, 13, 47]. According to one model, endocytosis of the LRP6 complex results in the sequestration of GSK3 in MVBs [48], thus protecting β-catenin from GSK3β triggered phosphorylation and proteosomal degradation. Another study showed that the acidic environment in endosomes facilitates Wnt ligand triggered LRP6 phosphorylation at T1479 [49]. Our findings are not consistent with this latter conclusion, since endocytosis defective LRP6-LDLRR mutants were phosphorylated at the T1479 residue.

We established that the Itch E3 ligase specifically associated with wild type LRP6 but not with our functionally impaired LDLRR mutants and that Itch was required for Wnt ligand triggered LRP6 endocytosis. How defects in the extracellular LRP6-LDLRR domain adversely affect the ability of LRP6 to interact with an intracellular Itch remain to be elucidated. Structural studies to date have focused on the LRP6 E1-E4 domains within the extracellular region [19-21], and hence, it is not yet known how its LDLRR domain or mutations therein may alter LRP6 conformation. As a monomer, LRP6 exhibits a horseshoe-like conformation with low density at the center of a compact structure [20]. Computational modeling of the LRP6 cytoplasmic region predicts that when inactive, this region is unstructured and may have a random coiled conformation [50]. Furthermore, this model indicates that molecules that interact with LRP6 at the cell surface may create spatial constraints that alter its intracellular conformation. The intracellular membrane proximal residues in LRP6 are palmitoylated and are required for its exit from the endoplasmic reticulum (ER) [51]. Moreover, palmitoylation is predicted to affect the orientation of LRP6 with respect to the cell membrane [51]. Thus, it is plausible that the LRP6-LDLRR mutants generated by us could cause interference with LRP6 binding to cell surface lipids in a manner analogous to the effects of FH LDLR mutations and adversely impact the ability of Itch to interact with the LRP6 cytoplasmic region. If so, LRP6 palmitoylation may affect its interaction with Itch.

Another ubiquitin ligase, zinc and ring finger 3 E3 (ZNRF3) mediates ubiquitination of the Wnt co-receptor Frizzled 8 and leads to its clearance from the cell surface through the proteosomal degradation pathway [52]. A Fzd lysine mutant that failed to be ubiquitinated by RNF43 did not internalize [53], supporting a requirement for Fzd receptor ubiquitination preceding its endocytosis. Moreover, the E-3 ligase, Mindbomb1, was shown to ubiquitinate Ryk, a Wnt co-receptor, and to facilitate its internalization to activate Wnt signaling [54]. Thus, the role of ubiquitination in regulating internalization of Wnt cell surface receptors in addition to LRP6 is well established.

Certain components of the Wnt pathway have bi-specific functions in modulating Wnt signaling. For example, GSK3β phosphorylates both LRP6 [6] and β-catenin [4]. Whereas GSK3β phosphorylation of LRP6 activates Wnt signaling [6], its phosphorylation of β-catenin inhibits Wnt signaling by targeting β-catenin for ubiquitin mediated proteosomal degradation [4]. Similarly, Axin overexpression inhibits Wnt signaling, but Axin is also required for the initial Wnt activation step involving LRP6 phosphorylation [9]. When Dvl, an important downstream Wnt component, is ubiquitinated by Itch, it is targeted for proteosomal degradation, which is inhibitory to Wnt signaling [55]. Thus, Itch too, has both positive and negative impact on Wnt targets, transiently activating the Wnt pathway by inducing LRP6 endocytosis and inhibiting the pathway by targeting Dvl, a downstream component for degradation, adding a new layer of complexity to the intricate regulation of Wnt signaling.

## METHODS

### Cell Culture

293T, L-Wnt3a and L-Control cells were purchased from American Type Culture Collection (ATCC) and cultured in Dulbecco's Modified Eagle's medium DMEM (Gibco) supplemented with 10% fetal bovine serum (FBS) (Sigma) and 1% penicillin and streptomycin (P/S) (Invitrogen). 0.4mg/ml G-418 (Gibco) was also added for culturing L-cells stably expressing Wnt3a. A3243 and A204 human sarcoma cells have been described previously [30]. U-2OS human osteosarcoma cells expressing inducible ubiquitin were provided by Dr. Zhijian J. Chen (University of Texas Southwestern Medical Center) and cultured as recommended [35]. Mouse embryo fibroblasts (MEFs) carrying floxed LRP5 and LRP6 alleles were provided by Dr. Bart O. Williams (Van Andel Research Institute). To generate LRP5−/−;LRP6−/− double knockout, MEFs carrying floxed alleles were transduced with adenovirus Cre recombinase (Viral vector core facility, University of Iowa) according to the recommended protocol.

### DNA constructs and site-directed mutagenesis

Flag tagged LRP6 wild type has been described previously [17]. Single base substitutions were generated in the LRP6-LDLRR using site-directed mutagenesis according to the manufacturer's protocol (Agilent). Lentiviral constructs for human LRP6 and D1315N-LDLRR were generated by amplifying the full-length fragments from pCMV-flag-LRP6-WT and pCMV-flag-LRP6-D1315N plasmids and inserted into the NSBI lentiviral vector backbone [30] using standard cloning procedures. Caveolin and LRP4 were purchased from Open Biosystems (GE Lifesciences). The Mesd full-length cDNA was synthesized from 293T cells and cloned in frame between EcoR1 and Not1 sites in pcDNA3 vector. His-S1317P LRP6 was generated by cloning in frame a His (6x) sequence CACCACCACCACCACCAC into HindIII and Not1 restriction sites in the pFlag-CMV vector. Flag sequences were mutated in two sequential steps using 5′GTTGGAGCTGCAGTTGCTGCCTACAAAGACGATGACGAC3′ and 5′GTTGAGCTGC AGTTGCTGCCTGCAAAGACGATGACGAC3′ primers by means of site directed mutagenesis (Agilent) to generate the pHis-CMV vector. S1317P LRP6 sequence from pflag-CMV-LRP6 was digested with Not1 and Xba1 and ligated into the same sites in pHis vector. Itch full length was PCR amplified from 501T cells and inserted between Nhe1 and EcoR1 cloning sites in the pcDNA3.1 vector (Invitrogen). C830 Itch mutant was generated by site directed mutagenesis (Agilent) using 5′GGCTACCCA GAAGTCATACCGCTTTTAATCGCCTGGACCTGCC3′ primers. HA-ubiquitin was obtained from Dr. Dirk Bohmann (University of Rochester). LRP6 KR10 was generated from pCMV-Flag-LRP6 by site directed mutagenesis. Itch (4390824) and scramble (4390843) siRNAs were purchased from Ambion. Lentiviral constructs for TCF-binding elements (TOP) (seven in tandem) luciferase reporter and constitutive Renilla luciferase were previously described [56]. Primer and other oligonucleotide sequences not listed here are available upon request.

### Real-time PCR

Total RNA was extracted using Trizol reagent (Invitrogen) according to the manufacturer's protocol. First strand cDNA was synthesized using random hexamers and Superscript11 reverse transcriptase (Invitrogen) following manufacturer's protocol. Human Axin2 and TBP primers [30] were used along with 50ng of cDNA as template in real-time PCR performed using SybrGreen PCR mix (Roche) as previously described[30].

### Transfection and viral transduction

All transfections were done using 1mg/ml Polyethylenimine pH7.2 (PEI: 24765-1; Polysciences). Lentiviral particles were generated in 293T cells by co-transfecting target vector, a packaging vector containing Gag, Pol, Rev, and Tat genes and an envelope encoding plasmid as described [30]. To generate MEFs expressing human LRP6-WT or D1315N LDLRR mutant, LRP5−/−;LRP6−/− double knockout cells were transduced with the respective lentiviruses and selected with 2.5ug/ml Blasticidin (Gibco). For siRNA transfection, RNAimax (Invitrogen) was used according to the manufacturer's protocol.

### TCF reporter assay

293T cells expressing the TCF/Wnt reporter were generated by transducing TOP-luciferase and Renilla-luciferase viruses in the presence of 10μg/ml polybrene (Sigma). Dual-Luciferase reporter assay kit (Promega) was used to measure reporter activity according to the manufacturer's protocol.

### Immunoprecipitation and Western blotting

For IP, cells were lysed in NP40 buffer containing proteinase inhibitors [30] and centrifuged at 13,000rpm for 20 min at 4°C to sediment the insoluble fraction. From the cleared lysates, 500ug-1mg of total protein was used for IP in the presence of 1:1000 dilution of primary antibody. After overnight incubation at 4°C, Protein G-sepharose beads (GE Lifesciences) were added and incubated for an additional 2 hrs. Beads were collected by spinning at 1500rpm and rinsed three times in NP40 buffer before resuspending in 2x laemmli loading buffer. Western blotting was performed as described previously [30]. Primary antibodies used were Flag (Sigma), LRP6 (IC10, Abcam), LRP5 (31E7, Abcam), Itch (Abcam), Caveolin (Abcam), pS1490 LRP6 (Cell Signaling), pT1479 LRP6 (Abnova), Rab7 (Cell Signaling), pT222 Itch (Abcam), α-tubulin (Sigma), HA (12CA5, MSSM hybridoma) and β-catenin (BD). Secondary antibodies tagged with either Alexa 680 or Alexa 750 fluors were from Invitrogen and used at 1:10,000 dilutions. Membranes were scanned using the Odyssey infrared imaging system (Licor).

### FACS

Around 36hrs post transfection with Flag tagged LRP6, 293T cells were rinsed twice with ice cold PBS. Cells were collected in Eppendorf tubes by gentle trypsinization using cell stripper solution (Cellgro) and briefly centrifuged. About 1-2 x 106 cells were resuspended in 100ul of PBS containing 1% FBS and 1:200 ratio of Flag (M2 Sigma). Cells were incubated on ice for 1 hour and then centrifuged briefly at 4°C. Cell pellets were rinsed twice with PBS and then resuspended in 100ul of PBS containing 1:1000 ratio of mouse Alexa 488 (Life Technologies). Cells were centrifuged and pellets were rinsed three times in PBS. After the final rinse, cells were resuspended in 500ul of PBS containing 1% paraformaldehyde. Data were collected by flow cytometry using a FACS Calibur (BD Biosciences) and analyzed on FlowJo data analysis software.

### Biotinylation assay

For internalization assay, 293T cells expressing flag tagged LRP6 were labeled first with 0.5mg/ml biotin (EZ-Link-Sulfo-NHS-SS-Biotin; Pierce) for 30 min at room temperature. Excess biotin was quenched with 100mM glycine (Sigma), and cells were treated with Wnt3a or control conditioned medium from L-cells at 37°C. Cells were gently scraped off the plates, rinsed in ice cold PBS three times, and treated with 50mM reduced glutathione (Fisher Scientific) dissolved in 75mM NaCl; 10mM EDTA, pH8 for 1hr on a rocker at 4°C. Cells were collected by brief centrifugation at 2000rpm and after rinsing three times with ice cold PBS were lysed in NP40 lysis buffer. 300-500ug of protein were immunoprecipitated with streptavidin beads (Pierce) and incubated for 1hr on a rocker at 4°C. Beads were rinsed three times with ice cold lysis buffer and resuspended in laemmli loading buffer for Western blot analysis[30]. For measurement of cell surface LRP6 levels, 293T cells expressing flag tagged LRP6 were treated with Wnt3a or control conditioned medium from L-cells at 37°C. Cells were rinsed three times with cold PBS and then treated with 0.5mg/ml EZ-Link-Sulfo-NHS-LC-Biotin (Pierce) for 30 min on ice. Samples were processed for immunoblotting as described for the internalization assay.

### Uncomplexed β-catenin assay

Uncomplexed β-catenin assay was performed as described previously [30]. Briefly, 1mg of protein from whole cell lysate was incubated with GST-E-cadherin/Glutathione-Sepharose beads (Amersham) for 1hr at 4°C. After the incubation, beads were collected by centrifugation and washed three times with NP40 containing lysis buffer and resuspended in laemmli loading buffer. Immunoblotting was performed as described above, and membranes were probed with β-catenin (BD) antibody.

### Mass spectrometry

293T cells growing in 10cm culture plates were transfected either with flag tagged hLRP6-WT, LRP6 S1317P or LRP6 D1315N using PEI. 48 hrs post-transfection, cells were treated with control or Wnt3a conditioned medium for 1 hr at 37°C. After the treatment, cells were lysed with NP40 buffer supplemented with complete proteinase inhibitors cocktail (Roche). Cleared lysates were used in IP with Flag M2-magnetic beads (Sigma). For mass-spectrometry analysis, pooled samples from 4 independent pull-downs were used. A total of about 7mg of protein was divided into two Eppendorf tubes, and 30ul of a 50% rinsed slurry of beads was added and incubated at 4°C overnight. The beads were then separated using a Dynamag magnetic separator Invitrogen), and rinsed 3x with 1 ml of NP40 buffer followed by 3x with 1ml of PBS. After the final rinse in PBS, bead associated proteins were eluted by adding 0.1M Tris Glycine, pH2.9 for 5 min at room temperature. Beads were separated and the eluate transferred to clean labeled Eppendorf tubes on ice followed by addition of 1M Tris.HCl, pH8 at 1/10th the total volume of the eluate. Sample processing and analyses were done at Yale University's Keck MS and proteomics core facility. Briefly, the LC-MS/MS data was acquired after trypsin and/or a dual Lys-C /trypsin digestion. Peptides were separated on a Waters nanoACQUITY (75 μm x 250 mm eluted at 300nl/min.) with MS analysis on a LTQ Orbitrap mass spectrometer. Mascot distiller and the Mascot search algorithm were used for database searching. Confidence level was set to 95% within the MASCOT search engine for protein hits based on randomness. For positive identification of proteins, two or more MS/MS spectra should match the same protein entry in the database searched. Proteins with only 1 significant peptide match are not considered positive identifications. Single peptide scores (i.e. only 1 peptide identified) of less than 20 were not used.

### Ubiquitination

293T cells were transfected first with HA-tagged ubiquitin using PEI, and 24 hrs later cells were trypsinized, pooled, divided equally and replated. Cells were retransfected with 2ug of flag tagged LRP6 constructs in the presence of empty vector control or Itch constructs. 36hrs later cells were treated with 5uM MG132 (Sigma) proteasome inhibitor for 5 hrs at 37°C. Cells were then scraped and transferred to Eppendorf tubes and lysed with 1% SDS and boiled for 5 min. After boiling, lysate was diluted 10 times with NP40 (no SDS) buffer. 500ug-750ug of cleared lysate was used in IP with Flag-M2 magnetic beads overnight at 4°C. After overnight incubation, beads were separated, rinsed three times with NP40 buffer and resuspended in laemmli protein loading buffer. Samples were run on a bi-gradient PAGE gel with 6% (top) and 12 % (bottom) concentrations. Proteins were transferred to a PVDF membrane and probed with Flag, HA, LRP6 and Itch primary antibodies. Except for Itch, the same membrane was reprobed.

### Statistical Analyses

Prism software was used to perform t-tests to determine statistical significance.

## SUPPLEMENTARY TABLE


